# Reply: A Correct FFR Trace Interpretation Is Important for a Clinical Decision

**DOI:** 10.1016/j.jaccas.2022.08.035

**Published:** 2022-10-19

**Authors:** Hakim Benamer

We have read with interest the remarks by Drs Chiabrando and Seropian regarding fractional flow reserve (FFR) measurements in our clinical case published in *JACC: Case Reports*^1^ and proposed advice for FFR measurements in general. We are in complete agreement with what is written.

Moreover, with regard to our published case,^1^ we had made several concordant measurements. The measurement proposed in Figure 1 of our paper^1^ made it possible to visualize the result at the top left at the same time as the curves. The remarks made are relevant to this image regarding the artifact, but the FFR before fistula occlusion was indeed at 0.8 and was checked several times. In addition, we see clearly in Figure 1 of our paper^1^ that the average of the FFR is 0.8 and stable, the scale being 0.2.

The patient’s clinical data seem to confirm the entire strategy, with angina and ischemia documented before occlusion of the fistula, followed by the disappearance of symptoms that has remained stable for several years.

More generally, it is true that FFR measurements must be carried out rigorously and repeatedly, especially when the result obtained is at the border of significance.
